# Dogmatism and Happiness

**Published:** 2017-03

**Authors:** Maryam MALMIR, Mohammad KHANAHMADI, Dariush FARHUD

**Affiliations:** 1. Research Center of Life Style, Medical Sciences Branch, Islamic Azad University, Tehran, Iran; 2. Aging Research Center, Scientific Cultural Foundation of Farhud, Tehran, Iran; 3. School of Public Health, Tehran University of Medical Sciences, Tehran, Iran; 4. Dept. of Basic Sciences, Iranian Academy of Medical Sciences, Tehran, Iran

**Keywords:** Subjective wellbeing, Happiness, Dogmatism

## Abstract

**Background:**

Happiness is a drive and constructive force of life. A person feels wellbeing under different effective factors. Religious dogmatism that has an influence on the entire world is one of the depreciatory factors of happiness or wellbeing. The current study decided to analyze the relation between dogmatism and wellbeing, and according to a model, answer the following question: how does religious dogmatism decrease wellbeing?

**Methods:**

This study is a correlation research. Population of study includes all people with 30–50 yr old who live in Tehran, Iran, in 2015. Among all, 180 subjects were selected as in access sample. The Oxford happiness questionnaire and Rokeach dogmatism scale were used. Data were analyzed by Pearson correlation test.

**Results:**

There is a significant negative correlation between dogmatism and happiness (α=0.05).

**Conclusion:**

Dogmatism is one of the factors that have a negative effect on wellbeing. Religious dogmatism is the most dangerous factor against wellbeing. Dogmatic individuals have an inflexible cognitive system that emerges as a stable personality trait and decreases their adjustment with environment. Affective well-being and cognitive wellbeing are affected by individual adjustment. Therefore, in dogmatic individuals with low adjustment, the decrease of affective well-being and cognitive wellbeing is inevitable. This process will result in decrease of happiness and increase of aggression.

## Introduction

Happiness is the fundamental factor in mental health. Achieving happiness was the earliest wishes of human being. Happiness points out to short-term effects and feelings and long-term well-being (1). In one point, happiness (regarded as subjective wellbeing in this article) is called as a heritable mood. In another point, happiness is seen as emotion and others see the happiness as cognitive evaluation. Therefore, it seems as a complex and controversial content. It includes positive emotions like; life satisfaction, optimism, sense of humor, forgiveness, tipsiness and so on. Philosophers and scientists describe several characteristics as critical criteria of pleasant life. They use several terms for happiness: eudemonia and virtues life, wellbeing, utopia, pleasant, high level of positive emotions, life satisfaction and so on.

Since appearance of positive psychology, happiness is studied as a major subject in scientific studies (2). The term “happiness” has many definitions. Each definition refers to a specific theory. Generally, all definitions divided into 4 categories: emotional-affective, cognitive, attitude and combined (3). The most common definition presented by Veenhoven; general judgment of a person about his/her quality of life as a whole (4).

Although there is no common construction for wellbeing, the similar basis can be traced in different cultures. Wellbeing consists of two factors: cognitive factors (life satisfaction) and emotional factors (hedonic level) (5).

The important thing about wellbeing is its underlying factors. Which factors do increase happiness or subjective well-being? Positive psychology’s researchers study various aspects of happiness and describe several indicating factors for happiness. A group of researchers believe that happiness results from genetic and heredity factors (6), another group believe that happiness results from earning high income (success in economic and job) (5), others believe that being able to live leads to happiness. Happiness is not the result of one or two factors; it is a combination of factors in a special way leading to happiness (5).

As a whole, indicating factors of happiness are divided into two groups: endogenic factors (genetic and biologic, cognitive, personality and ethical factors) and exogenic factors (behavioral, social, cultural, demographic, life event, geographic, political and economic factors) (2, 5).

Initial studies of wellbeing and happiness were focused more on exogenic (environmental) factors. Exogenic factors like; health, income, marriage, life events and so on, explain a little part of happiness. Studies conclude that since happiness is stable in time and after a major life event, it returns to base situation, happiness has a great significant correlation with endogenic factors like; personality traits (7,8).

A personality trait considered in relation with subjective wellbeing is the dogmatism. Dogmatism is a cognitive construction altered to a personality trait. Dogmatism has a significant negative influence on wellbeing. There is a negative relation between dogmatism and wellbeing (9). Dogmatism is defined as avoidance from accepting others’ beliefs, ideas and behaviors. Dogmatic individuals have many problems in understanding new ideas. They cannot accept reasonable ideas instead of their incorrect ideas. They do not cooperate with others with different ideas. They prefer to work with people like themselves. This group committed to their ideas without considering other possibilities (10, 11).

Dogma is a Greek word that means individual believe or idea. Individuals with open mind in acceptance of new ideas are without dogmatism and individuals with close mind present as dogmatism (12). An important theory about dogmatism was that dogmatism pointed to a cognitive network (13). Based on that, dogmatism can be attended in two levels.

“The first level, the isolation between and within belief and disbelief systems, is characterized by little differentiation within the disbelief system, isolation of parts within and between belief and disbelief systems, and high rejection of disbelief system. The second level that of the subordination of the peripheral beliefs to the central region of beliefs is characterized by the dependence-submission in an authoritarian way of the peripheral parts of beliefs to what constitutes the central beliefs.”(12)

In dogmatic person, the change in central region of beliefs affects the peripheral beliefs. In return, peripheral beliefs have no effect on central region of beliefs (14).

In this theory, dogmatism had three definitions:

1. A relatively closed cognitive system from beliefs and unbelief toward the reality,
2. Organizing a fundamental belief about absolute power,
3. Providing a framework of dogmatism forms towards everything (15).

In current societies, there are various forms of dogmatism that it is a challenge for the world. Dogmatism was developed mainly in following forms: political, racial, ethnic, religious, and so on. Dogmatism as a personality trait decreases the accommodation and it has negative effects on wellbeing. Therefore, the current study aimed to evaluate the relationship between dogmatism and wellbeing experimentally.

## Materials and Methods

This study was an applicable research and data were analyzed by correlation procedure. Population of study includes all people (30–50 yr) that come from Tehran, Iran, in 2015. Among all, 180 people with mean age 48 yr selected as an in access sample.

Ethical issues were attended for both selecting sample and performing the research. Demographic characteristics are presented in Table 1.

**Table 1:** Demographic characteristics of sample

**Group**	**Subgroup**	**Number**	**Percent**
Sex	Male	74	41
	Female	106	59
Age	30–40	98	54
	41–50	82	46
Education	Under-diploma	64	35
	Diploma	48	27
	Bachelor of art	31	17
	Master of art	28	16
	Ph.D.	9	5

For obtaining the data and assessing the variables, the following tools are used:

### Oxford Happiness Inventory (OHI) (7)

The OHI comprises 29 items, each involving the selection of one of four points (Likert scale) that are different for each item. The highest score on this scale is 87, which shows the highest point of happiness. Normal and mean range score on this scale is 40 to 42. Reliability of the OHI is 0.91 and internal correlation of items is about 0.04 to 0.67. In addition, reliability of the test in Iran by test-retest is 0.79.

### Rokeach dogmatism Scale

The 66 items form of Rokeach dogmatism scale was used. It is a valid and reliable scale. Reliability of the scale was assessed by test-retest method (0.69) (16). It is localized by researchers in Iran. Based on the expert’s report, validity of the scale was suitable and reliability of the scale is obtained about 0.71.

Data were analyzed by descriptive and referral statistics (Pearson correlation coefficient). Analysis of data was performed by SPSS-21 (Chicago, IL, USA).

## Results

Obtained data categorized in order and by using descriptive mathematics, mean and standard deviation is assessed for each variable. Mean and standard deviation of subjects in dogmatism (34.76, 11.75) and in happiness are (47.26, 15.22), respectively.

For assessing the relation between dogmatism level as an independent variable, with a dependent variable in research, Subjective well-being, the Pearson correlation coefficient is used (Table 2).

**Table 2:** Correlation coefficient between dogmatism & Subjective wellbeing

**Variable**		**Dogmatism**	**Happiness**
Dogmatism	Pearson correlation	1	−0.644^**^
	Sig. (2-tailed)		0.000
	N	180	180
Happiness	Pearson correlation	−0.644^**^	1
	Sig. (2-tailed)	0.000	
	N	180	180

**: Correlation is significant at the 0.01 level (2-tailed).

## Discussion

Dogmatism has negative effects on wellbeing. High levels of dogmatism lead to low level of happiness. Findings are in line with other studies (17, 18). Dogmatism has negative relationship with sense of humor (19).

This relationship can be explained by presentation of forming dogmatic thinking. One of the critical periods of life is the adolescence. In this stage of life, an adolescence encounter with identification challenge. Theoretically, different forms of identity (individual, social, ethnical, racial) develop in this period (20). Totally, identity pointed to awareness of a person about her/himself as an independent, unique and a person with special place in society (21). Attention to identity in psychology refers to the works of Erikson (1968). He explained in his book as “Identity: Youth and Crisis” identity is a critical challenge for each person (20).

Puberty is named in Erikson’s theory as “identity vs. role confusion”. Identity means essentially, how a person sees her/himself in relation to her/his world. It is a sense of self or individuality in the context of life and what lies ahead. Erikson believed that social groups have a clear role in forming identity (22). Membership of extremist groups and projection with these groups is the base of forming dogmatic thinking.

Another theory that designated to identity is that adolescence faces with four-identity status: achievement identity, moratorium identity, foreclosure identity, diffusion identity (Table 3).

**Table 3:** Kinds of identity (23)

**Position regarding career and ideology**	**Identity status**			
	Identity Achievement	Identity Moratorium	Identity Foreclosure	Identity Diffusion
Crisis Commitment	Passed crisis Present	In crisis Present but vague	Crisis absent Present	Crisis present or absent Absent

Each status results from a special combination of “commitment” and “crisis” (24).

Adolescences with foreclosure identity and diffusion identity have more problems in adaptation with environment. Dogmatism will be developed in families with rigorous thinking; they are all under control of parents. A person with foreclosure identity is more intended to dogmatism and inflexibility (25). Therefore, these children develop their cognitive networks based on their families’ forces and insist on acquisition rules.

Maladjustment and inability to accommodate with peripheral environment resulted from close mind and inflexibility against life events (25). Dogmatic person is unable to modify his cognition with new and challengeable events. They do not have an alternative solution for solving problems. They change most of problems based on limited acquired rules (26). Therefore, when they encounter a problem, they will experience frustration, and then they will avoid this problem (27, 28). Inflexible thinking has negative effects on adaptation through two ways:

1. Dogmatic people are unable to accept and understand opposite ideas. Violence is the predictive behavior when they encounter with challengeable events.
2. For inflexible forms of thinking, dogmatic individuals are unable to find different solutions in challengeable events. In another hand, they do not have creative and divergent thinking. Therefore, they have clear problems in adaptation with environment (26).

There are two dimensions for wellbeing: affective wellbeing, cognitive wellbeing (29, 30). According to two dimensions of subjective well-being, affective wellbeing, and cognitive wellbeing, the role of adaptation on each dimension should be considered. Affective well-being includes emotions and temperaments (negative and positive). Emotional theories suppose that negative emotions stimulate avoidance tendencies and positive emotions stimulate exposure tendencies. In contrast, temperaments are more affected by behavior (31).

Therefore, emotions and temperaments are a constant and accessible control system toward reaching goals. This system may be activated by some internal factors, but it would be back to the basic condition since it should be adopted by long-term changes (31, 32). Then, adaptation is an important function for affective well-being and adaptation is a necessary factor for balance of all system (33).

Changes in cognitive wellbeing may be fewer acts automatically. Cognitive wellbeing reflects self-evaluation about life. Major life events (if they have significant effects on goals, family, and job) have measurable and constant effects on cognitive wellbeing (34). Life events have more constant effects on cognitive wellbeing than affective wellbeing. For example, negative events affect both cognitive well-being and effective well-being, but the rate of effects on cognitive wellbeing is significant (31).

**Fig. 1:**
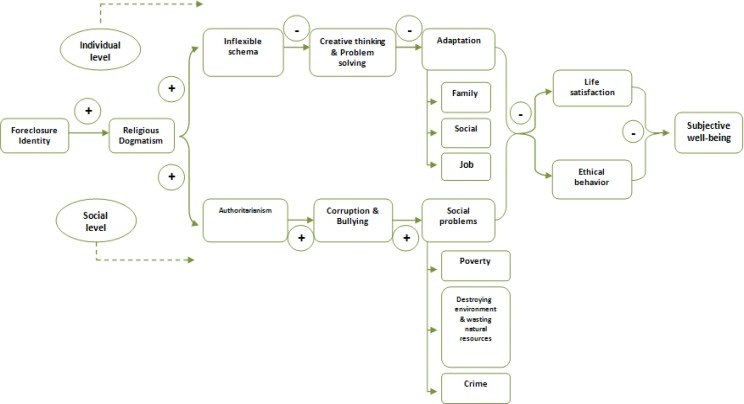
Impact of dogmatism on happiness

Since dogmatism decreases adaptation, dogmatic and close-minded people are not able to solve challenges, and they are not able to return to balance position, then their cognitive well-being and effective well-being is in danger (30). This danger is more in cognitive wellbeing than affective wellbeing. Happiness will be significantly decreased when cognitive wellbeing is decreased. So, inability of dogmatic people in adaptation with peripheral environment has negative effects on satisfaction and, therefore, it decreases the well-being or happiness (Fig. 1).

Dogmatism in social level has some consequences that it has negative effect on wellbeing. Dogmatism, especially religious dogmatism, associated with authoritarian, mythopoeia and individualism (35). Authoritarianism is an important factor for increasing immorality and bullying and results in social problems like poverty, gap, crime and destruction of natural resources. These social problems lead to decrease of wellbeing.

## Conclusion

Dogmatism has various subtypes: religious, racism, ethnic dogmatism. Although membership of a group increases social happiness, racism, and ethnic dogmatism decrease happiness. In addition, spiritual tendencies increase happiness, but religious dogmatism decreases happiness.

While there are various thoughts, ideas and believes in our world, dogmatism of any form (especially religious dogmatism) results from foreclosure identity and it decreases the individual adaptation with environment. Since they cannot accept the opposite attitudes and ideas, then they show maladjustment and violent behaviors. In addition, because of inability in creative thinking and in providing alternative solutions, they are missing ability of adjustment. While the critical factor for reaching happiness is adaptation with environment. Finally, inability of dogmatic people in adaptation with peripheral environment has negative effects on well-being or happiness.

## Ethical Consideration

Ethical issues (Including plagiarism, informed consent, misconduct, data fabrication and/or falsification, double publication and/or submission, redundancy, etc.) have been completely observed by the authors.
